# The role of artificial intelligence in developing a banking risk index: an application of Adaptive Neural Network-Based Fuzzy Inference System (ANFIS)

**DOI:** 10.1007/s10462-023-10473-9

**Published:** 2023-04-24

**Authors:** Ibrahim Elsiddig Ahmed, Riyadh Mehdi, Elfadil A. Mohamed

**Affiliations:** 1 College of Business Administration, Member of AI and DT Research Centers, Ajman University, Ajman, UAE; 2 Artificial Intelligence Research Center (AIRC), College of Engineering & Information Technology, Ajman University, Ajman, UAE

**Keywords:** Banking risk index, Artificial intelligence, Neuro-fuzzy systems, GCC banks

## Abstract

Banking risk measurement and management remain one of many challenges for managers and policymakers. This study contributes to the banking literature and practice in two ways by (a) proposing a risk ranking index based on the Mahalanobis Distance (MD) between a multidimensional point representing a bank’s risk measures and the corresponding critical ratios set by the banking authorities and (b) determining the relative importance of a bank’s risk ratios in affecting its financial standing using an Adaptive Neuro-Fuzzy Inference System. In this study, ten financial ratios representing five risk areas were considered, namely: Capital Adequacy, Credit, Liquidity, Earning Quality, and Operational risk. Data from 45 Gulf banks for the period 2016–2020 was used to develop the model. Our findings indicate that a bank is in a sound risk position at the 99%, 95%, and 90% confidence level if its Mahalanobis distance exceeds 4.82, 4.28, and 4.0, respectively. The maximum distance computed for the banks in this study was 9.31; only five out of the forty-five banks were below the 4.82 and one below the 4.28 and 4.0 thresholds at 3.96. Sensitivity analysis of the risks indicated that the Net Interest Margin is the most significant factor in explaining variations in a bank’s risk position, followed by Capital Adequacy Ratio, Common Equity Tier1, and Tier1 Equity in order. The remaining financial ratios: Non-Performing Loans, Equity Leverage, Cost Income Ratio, Loans to Total Assets, and Loans to Deposits have the least influence in the order given; the Provisional Loans Ratio appears to have no influence.

## Introduction

Artificial Intelligence (AI) in its various forms of machine learning, natural language processing and robotic process automation—is still in its early stage in terms of financial business applications.

Since the global financial crisis, risk evaluation and management in banks have gained more prominence, and there has been a constant focus on how risks are being detected, measured, reported and managed.

The use of AI by the banking sector is globally expanding as per the Global Association of Risk Professionals (GARP), and analytics leader SAS survey in 2018, the top area of AI use is the automation of manual processes (52%), followed by credit scoring (45%), data cleansing and enhancement (43%), risk grading (37%), model validation (35%), and model calibration (34%). Some newer-emerging AI applications with 20% or higher response rates were regulatory reporting, loan approvals, collections, and loan pricing.

All indications are that AI technology is here to stay and will become an increasingly important tool in risk monitoring, modeling and analytics. Risk professionals will likely have to broaden their abilities, melding domain expertise with highly quantitative and technical skills. Risk management departments may be re-skilled and reshaped, while quantitative and analytical capabilities are applied more comprehensively in more areas of the organization. McKinsey & Co highlighted that risk functions in banks, by 2025, would need to be fundamentally different from what they are today. The emergence of financial technology (FinTech) has seen a surge in interest and comment with regard to how AI might be developed and incorporated to better serve more traditional financial services and operations (Zhang and Kedmey 2018).

Himeur et al. (2022) explained that AI is facing many practical and operational challenges, ranging from the need for a basic familiarity with these systems to finding the necessary technical talent to managing the quality of big-data inputs and being able to understand and explain how AI models produce their outputs.

The review of the literature has shown that the application of AI (machine learning) in the management of banking risks such as credit risk, market risk, operational risk and liquidity risk has been explored by studies such as; Van Liebergen 2017; Deloitte University Press 2017; MetricStream 2018; Oliver Wyman 2017. However, all previous studies concentrated on individual components of banking risk rather than the overall ranking of all the risk measures as investigated by this study. A large number of areas remain in bank risk management that could significantly benefit from the study of how AI can be applied to properly address banking risk. Another motivation for this research is the need to consider all types of risk measures because of the increasing credit default and the negative impact of Covid 19 on banking performance and operations. In addition, most of the previous studies concentrated on the measurement of credit risk as the main source of default, but this study will be more comprehensive by including all other possible types and sources of risks such as operational, liquidity, market, and credit risks. This study will add great value to both limited literature and body of knowledge as well as to the banking practice through the ranking of risks variables.

A number of banking risk problems can be solved through the use of machine learning (ML). Measurement of risk and the analysis of key factors, including the study of the interconnections between the factors, can be achieved through the use of ML. For the purposes of developing a comprehensive risk index that considers all the banking risk areas and variables, Artificial Neural Networks (ANN), a genetic algorithm can be applied. ANN can be used in the approximation of the general risk trend and determination of the most influential risk variables. Therefore, the main objective of this study is to apply the most useful AI tool, Adaptive Neural Network-Based Fuzzy Inference System (ANFIS), to accurately compute, analyze, and develop an overall risk index of the Gulf banking sector. Furthermore, the study intends to explore the role of artificial intelligence in developing an index that ranks all the banking risk variables classified into their common categories as per their importance and influence on the banking operations and strategies.

The paper is organized as follows. Section 2 discusses the related literature while Sect. 3 covers the data and method. Section 4 presents the results and discussion. Finally, Sect. 5 provides a summary, conclusions and recommendations for future work.

## Related literature work

Models for financial distress forecasting of banks are being increasingly used as important tools to identify early warning signals for the whole banking system.

Numerous methods have been proposed for the analysis of banking risk problems, ranging from traditional methods to modern AI and machine learning techniques. To mention a few that include Neural Networks, Fuzzy Logic, Swarm Intelligence, Artificial Neural Networks (ANN) and Genetic Algorithms.

Mishraz et al. (2021) proposed a study that attempted to predict the financial distress of commercial banks by developing a bankruptcy prediction model for banks in India. In their study, they have developed three models using Logistic, Linear Discriminant Analysis (LDA) and Artificial Neural Network (ANN). The findings of the study indicate that the logistic and LDA models exhibit similar prediction accuracy. The results of the ANN prediction model exhibit better prediction accuracy. These findings clearly show the superiority of the AI techniques compared with the traditional ones.

In the same direction, another study that compared models developed using traditional methods and machine learning one to predict corporate bankruptcy is discussed in (Marso and EL Merouani 2020). In their study, they developed two models. The first model is multiple discriminant analysis (MDA), and the second one is an ANN trained by backpropagation (BPNN). The experimental results show that ANN models, on average, are approximately 10% more accurate in relation to MDA in different periods.

Another interesting survey on the applications of non-traditional techniques, such as AI on the forecasting of bankruptcy is given in (Aly et al. 2022). In this survey, they have compared the performance and accuracy of both traditional and non-traditional methods. Their result indicated that no single methodology can be the general superior tool for every bankruptcy situation. They have indicated that each methodology exhibits either strong or weak performance based on the situation and the used datasets.

A recent study that investigates corporate failure is discussed in (Jabeur et al. 2021). They have proposed a novel approach to classify categorical data using gradient-boosting decision trees, namely, CatBoost. Compared with eight reference machine learning models, their model demonstrates an effective improvement in the power of classification performance.

Researchers have studied the factors that have an impact on corporate failure. For example, González et al. (2021) proposed a Bayesian one-stage approach to estimate the effect of inefficiency on the time to failure (bankruptcy) of U.S. commercial banks. The result obtained show that their proposal outperforms the two-stage maximum likelihood approach traditionally used in the literature. In addition, empirical evidence suggests that the inefficiency of U.S. commercial banks during the global financial crisis in 2008–2009 played a statistically and economically significant role in determining the time to failure.

The neuro-fuzzy system, one of the AI techniques, has vast applications in business which include stock market prediction (SU and Cheng 2016; Vlasenko et al. 2019; Mohamed et al. 2021).

Rajab and Sharma (2018) presented a review of the applications of Neuro-Fuzzy Systems (NFS) in business on the basis of the research articles issued in various reputed international journals and conferences during 2005–2015. The result of this survey indicated that finance is among the viable application of Neuro-Fuzzy systems.

An interesting study conducted by Brlečić Valčić (2021) that used Adaptive Neuro-Fuzzy Inference System (ANFIS) approach, to produce models for investigating the effect of cost structure on sustainable development and business persistence with respect to selected financial indicators. Before building the model using clustering method, Brlečić Valčić (2021) established a link between the business performance indicators and parameters that increase or decrease a particular cost component.

Other AI methods used for the analysis of corporate financial distress include Interval-valued Fuzzy cognitive maps (IVFCMs) to model additional uncertainty in decision-making tasks with complex causal relationships (Hajek and Prochazka 2018). Hajek and Prochazka (2018) introduced a novel IVFCM with real-coded genetic learning. They have demonstrated that the proposed method is effective for predicting corporate financial distress based on causally connected financial concepts. In comparison with other methods, the proposed one outperforms Fuzzy cognitive maps, fuzzy grey cognitive maps and adaptive neuro-fuzzy systems in terms of root mean squared error.

Neuro-fuzzy also has been used in predicting the future stock price value. For example, Vlasenko et al. (2019) proposed a novel ensemble neuro-fuzzy model used to address the limitations and improve the previously successfully applied a five-layer multidimensional Gaussian neuro-fuzzy model and its learning. The suggested solution allows skipping the error-prone hyperparameters selection process and shows better accuracy results in real life financial data.

Another example of the use of Neuro-fuzzy in stock price prediction is given in (Su and Cheng 2016). In their study they have proposed a novel ANFIS (Adaptive Neuro Fuzzy Inference System) time series model based on integrated nonlinear feature selection (INFS) method for stock prediction. In their proposed model an integrated nonlinear feature selection method to select the important technical indicators objectively has been suggested. Next the method used ANFIS to build time series model and test forecast performance, then utilized adaptive expectation model to strengthen the forecasting performance. The performance evaluation of the proposed model using the TAIEX and HSI stock market transaction data outperformed the listing models in accuracy, profit evaluation and statistical test.

The surveyed literature indicates that there are many built models that aim to predict corporate bankruptcy. These models ranged from traditional one to the recent AI and machine learning. The survey results clearly indicate that the performance of the AI and Machine learning in bankruptcy prediction are better than the traditional one. Kar et al (2022) state that the Lack of an AI adoption strategy and lack of AI talent are the most significant barriers to AI applications.

Over the last decade, there has been a plethora of works in the literature in regard to corporate and bank bankruptcy prediction using AI and machine learning methods. However, researches exploring the application of ANFIS for banks bankruptcy prediction and the identification of banking risk index are scarce. The only work that uses neuro-fuzzy system for bankruptcy prediction we found is discussed in (Hajek and Prochazka 2018). Some studies applied AI techniques to recognize and predict human actions and activities (Gupta et al. 2022; Neu et al. 2022) focused on deep learning.

### Risk areas and variables

#### Capital adequacy risk

Adequate capital base acts as a financial safety against a variety of risks a bank is exposed to in its daily operations. Capital adequacy reflects whether the bank has enough capital to absorb unanticipated losses and declines in asset values that could otherwise cause a bank to fail, and provide protection to depositors and debt holders in the event of liquidation. The balance sheet of the bank cannot be expanded beyond the level determined by the capital adequacy ratio (CAR). The important parameters which provide an insight into the capital adequacy of the bank that are used in the current study are (i) Tier1 ratio, (ii) Total capital ratio.

(i) Tier 1 Capital Ratio compares a bank's core equity capital to total risk-weighted assets. A firm's core equity capital is known as its Tier 1 capital and is the measure of a bank's financial strength based on the sum of its equity capital and disclosed reserves, and non-redeemable, non-cumulative preferred stock. A firm's risk-weighted assets include all assets that the firm holds that are systematically weighted for credit risk. Basel committees recommend the weights which are further modified by the central banks, if required, for different asset classes, such as cash and coins, which have zero risk, versus a letter or credit, which carries more risk. A Bank must have a Tier 1 capital ratio of 8% (as per Basel guidelines) or greater. More than 8% is indicator of good risk position.
(ii) Total capital ratio (CAR): Total Capital ratio or Capital adequacy ratio is the ratio which determines the bank's capacity to meet the time liabilities and other risks such as credit and operation risk (Yang et al. 2020). Banks are required to maintain this ratio which is supposed to provide "cushion" for potential losses, and thereby protects the bank's depositors and other lenders. In this ratio two types of capital are measured: tier one capital, which can absorb losses without a bank being required to cease trading, and tier two capital, which can absorb losses in the event of a winding-up and so provides a lesser degree of protection to depositors. Basel stated a minimum acceptable ratio of 12%.
(iii) Common Equity Tier 1 Capital (CET1): Common Equity Tier 1 Capital is the highest quality of capital available reflecting the permanent and unrestricted commitment of funds that are freely available to absorb losses. It essentially includes ordinary share capital, retained earnings and reserves less prescribed deductions. The Basel standard is 10% and above.

#### Credit risk

Credit can be defined as the risk of potential loss to the bank if a borrower fails to meet its obligations (interest, principal amounts) (Thomas et al. 2022; Elsiddig and Sara 2015). Credit risk is the single largest risk banks face (Apostolik et al. 2009). The Basel Accord allows banks to apply the internal ratings-based approach to assess credit risk. They can internally develop their own credit risk models for calculating expected loss. As lending is the primary business for the banks. Asset quality is an important parameter to test the financial credibility of the banks and their risk exposure. Asset quality can be tested using different measures such as (i) Non-Performing loans to gross loans, (ii) loan loss provisions to net interest revenue.

(i) Non-performing Loans to gross loans (NPL/Loans): This ratio is an important sub-parameter to measure asset quality of the bank as it determines the loan quality. Higher the ratio more problematic the loans are and vice versa. A decreasing trend for this ratio over the years is desirable, as it indicates that the Banks are following more cautious approach to risk management and there is a fall in the problem loans.
(ii) Loan loss provisions to net interest revenue (Loan LP): loan loss provisions are set aside by banks as an allowance against bad loans. This ratio clearly indicates the quality of the existing loans thereby the financial strength of the banks. The lower ratio is more preferable as it stands for less loss as compared to the interest revenue.

#### Liquidity

Liquidity is the ability of the bank to meet financial obligations as they become due, without incurring unacceptable losses (Chen 2022). Liquidity corresponds the bank’s ability to make payments to its customers punctually; their inability to make these payments will detrimentally affect the bank’s solvency. Liquidity management for a bank extends to both its loan customers and depositors. Since fractional reserve banking means that banks keep only a fraction of their deposits available for immediate withdrawal, improperly managing the bank’s liquidity risk could lead to serious consequences like liquidity crisis which may lead to bank run.

(i) Net Loans / Total Assets (Loan/TA): The ratio of net loans to total assets indicates what percentage of the assets of the bank are tied up in loans. The higher the ratio the less liquid the bank is. A low ratio of loans to deposits indicates excess liquidity, and potentially low profits, compared to other banks. A high loan-to-deposit ratio presents the risk that some loans may have to be sold at a loss to meet depositors' claims.
(ii) Net Loans / Total Deposits (Loan/Dep): This ratio measures the degree of illiquidity of the bank as it indicates the percentage of the total deposits which are locked into non-liquid assets. A high figure denotes lower liquidity and high risk.

#### Earning quality risk

Earning quality ratios are used to measure the ability of the bank to earn profit compared to expenses. It shows the bank's overall efficiency and performance as it examines the bank’s investment decisions as compared to their debt situations. The Sub-parameters chosen to measure earning quality in this study are (i) net interest margin, (ii) financial risk.

(i) Net Interest Margin (NIM): The core functions of banks are accepting deposits and lending. For that reason, net interest margin acts as a prime ratio in measuring the bank’s performance because it is the difference between what they receive from what they pay. Bank management is expected to keep a stable net interest margin as it illustrates the extent to which bank is exposed to interest rate fluctuation and also reflective of bank’s management’s ability to effectively manage interest rate risk. A positive and high ratio is considered to be desirable as it implies the bank had made optimal lending decisions and is successful in getting the timely interest on loans back from the customers.
(ii) Financial Risk (LEV): This ratio reflects the risk associated with the sources of finance and capital structure. Reliance on debt finance results in high leverage ratio and stands for high risk, whereas more equity finance as compared to total assets is a sign of low risk and is a base for generating more earnings from the internal sources of finance. This ratio has a positive relationship with the degree of financial risk and earnings quality.

#### Operational risk

Operational risk is defined by BCBS as the risk of loss resulting from “inadequate or failed internal processes, people and systems or from external events” and is a “fundamental element of risk management” at banks. This definition includes legal risk, but excludes strategic and reputational risk (Roumani et al. 2020). It is considered inherent in all banking products, activities, processes and systems (Basel Committee on Banking Supervision 2011). The best financial measure of it is through the comparison between cost and generated income.

(i) Cost to income ratio: This ratio is an operating measure as it indicates the bank’s ability to manage its cost against income. Minimizing cost against income denotes efficiency hence lower the ratio higher is the efficiency. It measures how costs are changing compared to income. The cost income ratio, defined by operating expenses divided by operating income, can be used for benchmarking by the bank when reviewing its operational efficiency. Due to the inverse relationship between the cost income ratio and the bank's profitability, highly efficient banks will have low ratio and they generate higher profits.

## Data and methodology

The data on different types of risks is collected from the banks operating in the Gulf Council Countries (GCC). The data set covers the last five years (2016 to 2020). The main source of this secondary data is the financial statements and reports of the GCC banks available on the DataStream and the banks’ websites.

Bostrom (2014), stated that a full AI solution would be automated in terms of data identification, data testing, and making decisions based on the data testing. In practice, AI might involve additional techniques in addition to ML, such as including hard-coded and logic rules. ML on the other hand normally involves manual data identification and testing by the data scientist, and human decisions as to how to apply the outputted information. Given the lack of technological and organizational readiness for pure AI, and the reality that most claimed AI is in fact ML, the study will apply ML to risk measurement, analysis, and prediction. The study will test the accuracy of the measures of different types of banking risk and develop an overall measure of the risk, then will use ML to develop a model for predicting banking risk.

Some of the recent studies have revealed that emerging artificial intelligent techniques, such as Decision Tree (DT), Support Vector Machine (SVM), Genetic Algorithm (GA) and Artificial Neural Networks (ANN) are advantageous to statistical models and optimization technique for credit risk evaluation. In contrast with statistical methods, AI methods do not assume certain data distributions. These methods automatically extract knowledge from training samples. According to previous studies, AI methods are superior to statistical methods in dealing with corporate credit risk evaluation problems, especially for nonlinear pattern classification.

### Dataset

Ten financial ratios of 45 Gulf banks were collected for a 5-year period resulting in a dataset, Gulf Banks, consisting of 223 observations. As shown in Table 1, the ten financial ratios are: Tier 1 Equity Ratio (TEIR1), Capital Adequacy Ratio (CAR), Common Equity Tier 1 Ratio (CET1), Provisional Loans Ratio (PLoans), Non-Performing Loans Ratio (NPLoans), Loans to Total Assets Ratio (LTAR), Loan Deposit Ratio (DepLoans), Equity Leverage Ratio (EQLEVR), Net Interest Margin Ratio (NIMR), Cost Income Ratio (CIR).

**Table 1:** Risk categories, variables, and measures

Risk categories	Variable	Measure (Ratio)
Capital Risk	CAR	(Tier1 + Tier II) / TA
	TIER 1	Core Capital/Risky Weighted Assets
	CET1	Equity Tier 1/TA
Liquidity Risk	Loan to Deposit (DepLoans)	Loan/Total Deposits
	Loans Assets Ratio (LTAR)	Loans/TA
Operational Risk	Efficiency (CIR)	Cost / Income
Credit Risk	Provision Loan Ratio (PLoans)	Provision
	Non-Performing Loans (NPLoans)	NPL/Loans
Earnings Quality EQ	Net Interest Margin (NIMR)	NII/NI
	Leverage (EQLEVR)	(Assets-Liab)/TA or Equity/TA

The risks variables have been assigned as measures for the following 5 categories of risk.

X1 = CAPR is capital risk.

X2 = Credit risk.

X3 = LIQR is liquidity risk.

X4 = EQ Risk is earning quality risk.

X5 = OPR is operational risk** = **Cost/income.

### Proposed risk ranking index metric

Based on the Basel Accords and GCC Central Banks’ requirements for the minimum and maximum ratios, we have established a 10-dimensional point that reflects the minimum value for each financial ratio; the maximum ratios were replaced by their inverse. We referred to this point as the “critical risk ratios” (CRR) as it represents the minimum ratios that a bank must adhere to ensure survival. The more the distance between the point representing the financial ratios of a bank from CRR, the better the bank’s financial position and the less the risk of its insolvency. The recommended values of the ten financial ratios used for the critical point are: TIER1, 8.5 min; CAR, 10.5 min, CET1, 7.0 min; PLoans, 2.5 max; NPLoans, 5.6 max; LTAR, 70.0 max; DEPLoans, 85.0 max, EQ_LEVR, 3.5 min, EQ_NIM, 0 min; CIR, 50.0 max.

In this work, we have chosen to use the Mahalanobis distance (MD) as a measure of the Risk Ranking Index (RRI) as an indicator of a bank’s financial position. The MD is a measure of the distance between a multi-dimensional point (P) and a distribution of such points (D) (Mahalanobis 1936). It is a multi-dimensional generalization of the distance between P and D measured in terms of the number of standard deviations. This distance is zero for P at the mean of D and grows as P moves away from the mean along each principal component axis. If each of these axes is re-scaled to have unit variance, then MD corresponds to standard Euclidean distance in the transformed space. The MD is thus unitless, scale-invariant, and considers the correlations of the data set (Hadi and Simonoff 1993; Hill et al. 2006). In addition, the Mahalanobis distance is commonly used to identify outliers (Hadi and Simonoff 1993). The RRI indicator for a particular bank is computed as the MD between the multidimension point of the financial ratios of that particular bank and the CRR point. A bank is in a good financial position if it appears as an outlier with respect to the CRR point. A confidence level, such as, 95%, is used to construct the outlier’s boundary.

The Mahalanobus distance is given by Eq. (1) [ Hadi and Simonoff 1993]:

1 $$D^{2} = \left[ {\left( {X_{p1} - X_{p2} } \right)^{T} \times C^{ - 1} \times \left( {X_{p1} - X_{p2} } \right)} \right]^{0.5} ,$$

where *X*_*p1*_*, X*_*p2*_ is a pair of multidimensional points, and *C* is the sample covariance matrix

**Fig. 1:**
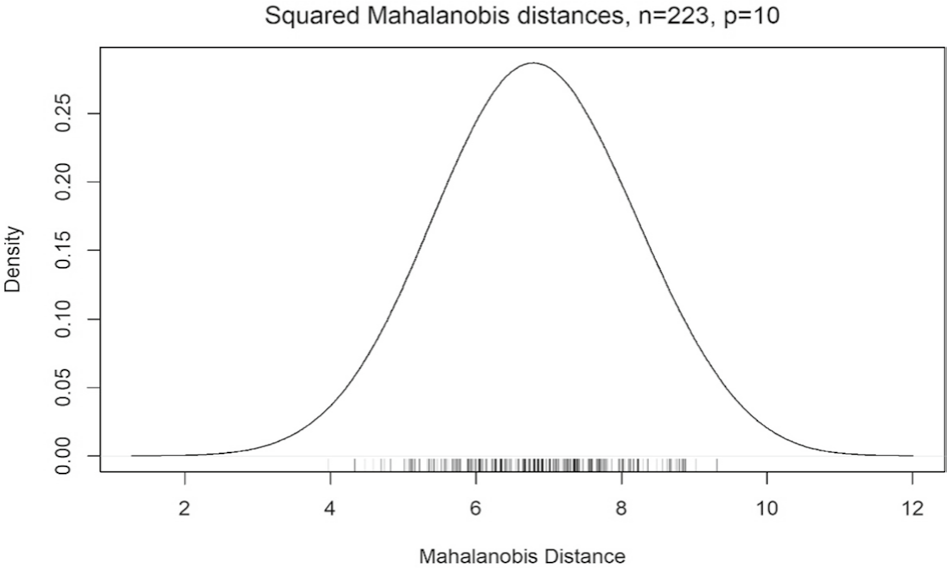
Distribution of the distance from the critical point

Using the R programming language, we have computed the distance of each multidimensional point representing the ten financial ratios from the CRR point. Figure 1 shows the distribution of the distance values with the following statistics: mean = 6.81, standard deviation = 1.046, minimum distance = 3.96 maximum distance = 9.31. The distance computed represent the relative strength of the financial position of the bank with respect to the critical point, CRR. Thus, the bank with maximum distance has the strongest financial position and the reverse is true for the bank having the minimum distance. The cut-off distance (outliers boundary) from the CRR point, at the 95% confidence level, was found to be 4.28. There is only one instance where a distance of 3.96 was found to be less than the cut-off distance, yet close to the outliers’ boundary indicating that the bank still has low risk of going insolvent. In fact, at the 85% confidence level, the cut-off point is 3.81, and all banks are sufficiently distant from the critical point.

**Fig. 2:**
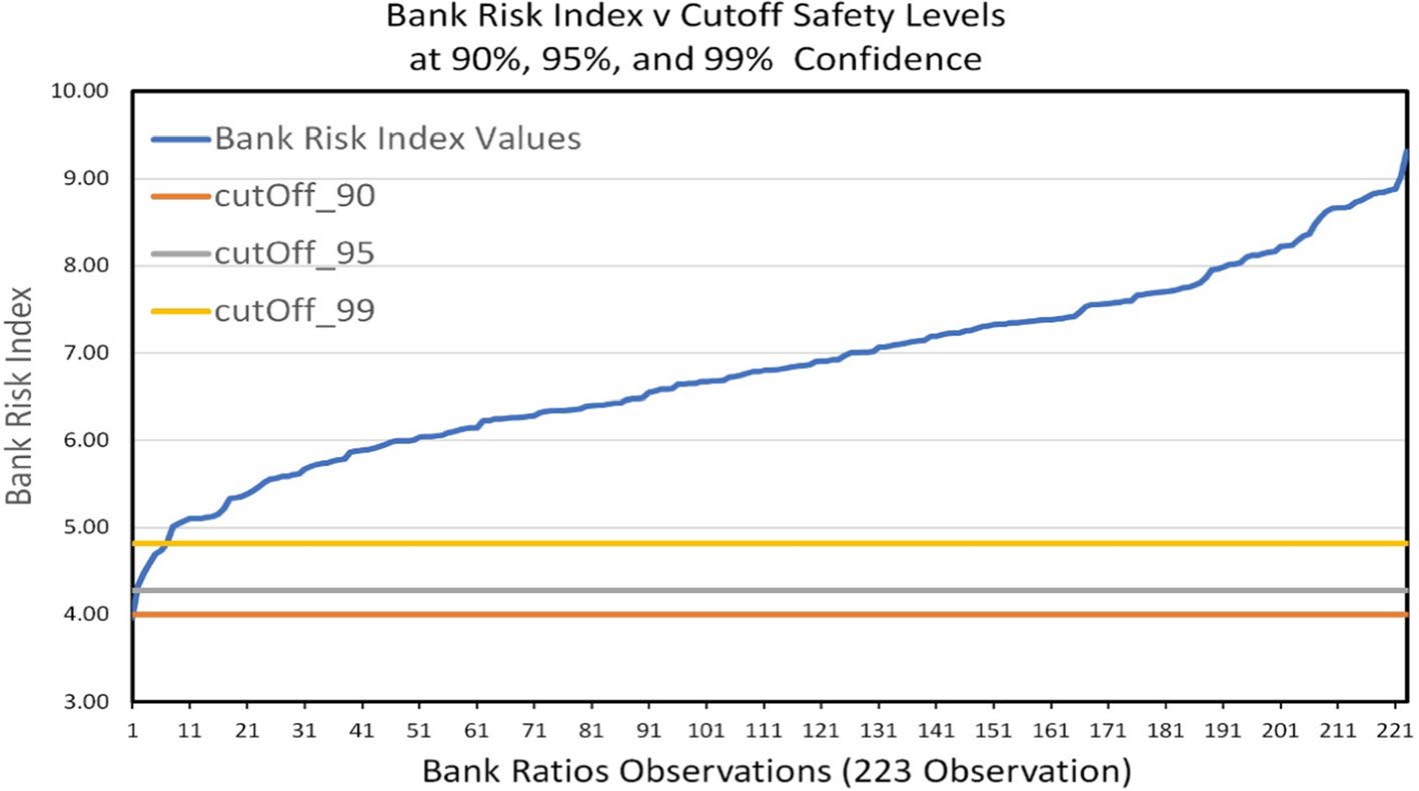
Bank risk index as measured by the Mahalanobis distance and cutoff distances from the critical point at 99%, 95%, and 90% confidence levels

A bank is in good standing with respect to risk if its multidimensional point representing its financial ratios is an outlier with respect to its distance from the critical point. The cutoff distance that classifies a point as an outlier can be computed from the Chi-squared distribution based on the confidence level required and the degrees of freedom measured by the number of input variables. Figure 2 shows the bank risk index as measured by the Mahalanobis distance for the 233 financial ratios analyzed in this work and the cutoff distances of 4.82, 4.28, and 4.0 at the 99%, 95%, and 90% confidence levels, respectively. Five cases were identified to be below the cutoff distance of 4.82 at the 99% confidence level, and one case was below the 95% and 90% levels with a distance of 3.96. No case was found below the cutoff distance at the 89.5% confidence level. Thus, the best financial ratios are those having a Mahalanobis distance of at least 4.82 from the critical financial ratios.

### Variables influencing the risk ranking index

In order to conduct a sensitivity analysis to determine the relative importance of the ten financial ratios in Table 1, we need to fit a model that captures the functional relationship between the RRI and the ten financial ratios. In this work, we have opted to use ANFIS to build the model. ANFIS is a hybrid analytical method that combines the merits of the neural network and the theory of fuzzy logic systems in its prediction mechanism (Jang 1993; Negnevitsky 2017). While neural networks control the representation of information and the physical architecture of the model, fuzzy logic systems imitate human reasoning and increase the model’s ability to manage uncertainty within the system (Jang 1993; Negnevitsky 2017). ANFIS basically learns the features of a given data and alters the system parameters to suit the required error criterion of the system in order to generate an output. Thus, in order to avoid making any assumptions with regard to the complexity, uncertainty, and the linearity or otherwise of the cause effect relationship between the Risk Ranking Index (RRI) and its determinants an attempt is made in this paper to develop ANFIS-based model for developing a model describing the relationship between the input variables (financial ratios) and the RRI computed as the Mahalanobus distance rather than opting for conventional methods such Multiple Linear regress (MLR) which basically estimates the parameters of a predetermined functional form of the relationship between the dependent and independent variables (Pal and Bharati 2019).

Table 2 shows the critical, best, and worst financial ratios identified in this work for banks working in the Gulf region. Some of the ratios in the worst case violate the specified ratios to keep the bank in a sound financial position. The Non-Performing Loans are just above the maximum under the worst instance with 5.8 compared to the critical ratio of 5.60. In addition, Loans to Assets and Loans to Deposits have obviously violated their maximum critical ratios with the worst scores of 85.5 and 93.2, respectively.

**Table 2:** Best and worst financial ratios for banks working in the Gulf region

Input financial ratio	Critical financial ratios	Best financial ratios instance (Risk Index 9.31)	Worst financial ratios instance (Risk Index 3.96)
TIER1_Limit	Min: 8.5	16.86	14.90
CAR_Limit	Min: 10.5	16.90	15.30
CET1_Limit	Min: 7.0	15.30	12.00
LoansLP_Limit	Max: 2.5	1.66	0.13
LoansNPL_Limit	Max: 5.6	5.11	5.80
LoansTA_Limit	Max:70.0	62.80	85.50
LoansDep_Limit	Max: 85.0	79.70	93.20
EQ_LEV_Limit	Min: 3.5	49.70	11.60
EQ_NIM_Limit	Min: 0	3.20	1.70
CI_Ratio_Limit	Max: 50.0	32.40	44.30

Fuzzy system models fall into two categories, which differ fundamentally in their abilities to represent different types of information. The first category includes linguistic models, which have been referred to as Mamdani fuzzy models. They are based on collections of if–then rules with vague predicates and use fuzzy reasoning (Mohamed et al. 2021). In these models, fuzzy quantities are associated with linguistic labels, and a fuzzy model is essentially a qualitative representation of the underlying system (Tanaka and Sugeno 1998). The second category of fuzzy models is based on the Takagi–Sugeno (TS) method of reasoning (Terano et al. 1994; Yager and Filev 1994; Sproule et al. 2002; Babaei and Bamdad 2020). These models are formed by logical rules that have a fuzzy antecedent part and a functional consequent. Fuzzy models based on the TS method of reasoning integrate the capability of linguistic models for qualitative and quantitative information representation (Rutkowski 2004). The main difference between Mamdani and Sugeno fuzzy systems is that the Sugeno output membership functions are either linear or constant. A Sugeno Fuzzy Inference System (FIS) is superior to a Mamdani FIS with regard to computational efficiency, accuracy, robustness in the presence of noisy input data (Subhedar and Birajdar 2012; Hamam and Georganas 2008; and MathWorks 2020). In addition, Sugeno FIS works well with optimization and adaptive techniques, guarantees continuity of the output surface, and well suited to mathematical analysis. As a result, this work uses ANFIS methodology based on a Sugeno FIS model. A description of the model structure and learning mechanism is provided in the subsections below.

### Adaptive neural network-based fuzzy inference system

A shortcoming of fuzzy systems is the lack of ability to learn and adapt to changes in their environment. The opposite is true with neural networks, they can learn from data, but their reasoning is embedded in the connection weights of their neurons (Mitra and Hayashi 2000). The integration of fuzzy inference systems and neural networks can provide a platform to build intelligent systems by replacing the weakness of one system by the strength of the other (Negnevitsky 2017). Jang (1993) proposed a neural network that is functionally equal to a Sugeno fuzzy inference model called the Adaptive Neural Network-Based Fuzzy Inference System (ANFIS) that integrates the mechanisms of neural networks and fuzzy inference systems to utilize the capabilities of both.

#### ANFIS architecture

**Fig. 3:**
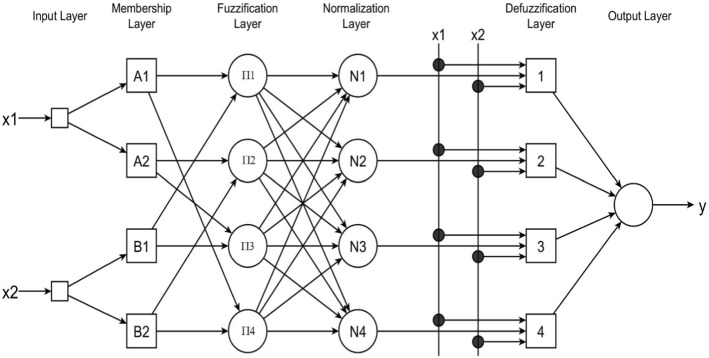
An adaptive Sugeno neuro-fuzzy inference system Architecture

Jang’s ANFIS (1993) is normally represented by a six-layer feedforward neural network. Figure 3 below shows the ANFIS structure that corresponds to a first-order Sugeno fuzzy model with two inputs and two membership functions per input from Negnevitsky (2017). The network has fixed and adaptive types of neurons. Fixed neurons are represented as a circle and the adaptive ones depicted as a square. The following exposition is adapted from Negnevitsky (2017).

For a first-order Sugeno fuzzy model, a two-rule base is expressed as follows:

2 $$Rule1:if\;x\;is\,A_{1} and\;y\;is\;B_{1} ,then\;f_{1} = p_{1} x + q_{1} y + r_{1} ,$$

3 $$Rule2:if\;x\;is\;A_{2} \;and\;y\;is\;B_{2} ,then\;f_{2} = p_{2} x + q_{2} y + r_{2} .$$

Gaussian, triangular, sigmoidal, S-shaped, among other membership functions, can be used with an ANFIS. Sugeno neuro-fuzzy algorithm performs best when used with a Gaussian type membership function. With Gaussian fuzzy sets, the algorithm is capable of utilizing all information contained in the training set to calculate each rule conclusion unlike using triangular partitions (Jain and Martin 1998). Sambariya and Prasad (2017) have found that a Gaussian membership function performs best when the number of membership functions is 3 or 5.

Let the membership functions of fuzzy sets *A*_*i*_, *B*_*i*_ for *i* = 1, 2 be two Gaussian membership functions *μ*_*Ai*_ and *μ*_*Bj*_ respectively. In evaluating the rules, a product T-norm (logical *and*) is chosen. Evaluating the rule premises using product T-norm results in:

4 $${w}_{i}={\mu }_{{A}_{i}}\left(x\right){\mu }_{{B}_{i}}\left(y\right), i=\mathrm{1,2}$$

Evaluating the implication and the rule consequents give:

5 $$f\left(x,y\right)=\frac{{w}_{1}\left(x,y\right){f}_{1}\left(x,y\right)+{w}_{2}\left(x,y\right){f}_{2}\left(x,y\right)}{{w}_{1}\left(x,y\right)+{w}_{2}\left(x,y\right)}$$

Leaving the arguments out:

6 $$f=\frac{{w}_{1}{f}_{1}+{w}_{2}{f}_{2}}{{w}_{1}+{w}_{2}}$$

The above equation can be rewritten as:

7 $$f={\overline{w}}_{1}{f}_{1}+{\overline{w}}_{2}{f}_{2}$$

where,

8 $${\overline{w}}_{i}=\frac{{w}_{i}}{{w}_{1}+{w}_{2}}$$

#### ANFIS learning

ANFIS uses a combination of least-squares estimator and the gradient descent algorithm to learn its parameters (Jang 1993). Initially, activation functions are assigned to each membership function neuron. The centres of the membership functions are set so that the range of input is divided equally, and the widths and slopes are set to allow sufficient overlapping of the respective functions (Negnevitsky 2017). For each epoch, training is conducted in two steps: forward pass and a backward pass. In the forward pass, the ANFIS learning mechanism uses training patterns to estimate the parameters of the consequents of the rules by a least-squares algorithm. Once the rule's consequent parameters are established, the network can compute the error. In the backward pass, the errors are propagated back, and the parameters of the membership functions are adjusted using the back-propagation learning algorithm (Negnevitsky 2017). In the ANFIS training algorithm suggested by Jang (1993), both antecedent parameters and consequent parameters are optimized through the learning process. In the forward pass, the consequent parameters are adjusted while the antecedent parameters remain fixed. In the backward pass, the antecedent parameters are modified while the consequent parameters are kept fixed. Membership functions can be described by a human expert and kept fixed throughout the training process when the input–output data set is relatively small (Negnevitsky 2017).

### Programming environment

The following Matlab functions have been used to implement the Sugeno type neuro-fuzzy system (MathWorks 2020):

1. *fismat* = *genfis(trnDataInput, trnDataOutput, optGenfis)*, genfis generates a fuzzy inference system using fuzzy c-means (fcm) clustering to extracting a set of rules that model the data behavior with the function *fcm()*. The function *fcm()* determines the number of rules and membership functions for the antecedents and consequents. The arguments for genfis are as follows (Sambariya and Prasad 2017; Talpur 2022):

- *trnDataInput*: a matrix where each row contains the input values, financial ratios, of a data point. The matrix *trnDataInput* has one column per input variable.
- *trnDataOutput*: a matrix where each row contains the output values of a data point. In this work, each input vector has one output, *Risk Ranking Index*, and thus *trnDataOutput* is a column vector.
- *optGenfis* = *genfisOptions(clusteringType),* creates a default option set for generating a fuzzy inference system structure using *genfis()*. In this work, we have used subtractive clustering to find the cluster centres.

2. The model has been built using k-fold cross validation data (k = 5) in building the fuzzy inference system. To enforce the use of validation data the **anfisOptions** function was used to set the **ValidationData** option: optAnfis = anfisOptions('InitialFIS', fismat, 'ValidationData', valData);
3. *fismat1* = *anfis(trainingData, optAnfis),* this function fine-tune a Sugeno-type fuzzy inference system, fismat, using training data and optAnfis generated by anfisOptions(). The options allow the user to specify: an initial FIS object to tune; validation data to prevent overfitting to training data; and training algorithm options such as *EpochNumber, ErrorGoal, InitialStepSize, StepSizeDecreaseRate, StepSizeIncreaseRate, OptimizationMethod* among others (MathWorks 2020). Default values are used for options not overridden.
4. *predictedOutput* = *evalfis(inputData, fismat1)* uses the fuzzy inference system *fismat1* and test data as input data to predict the bank’s *Risk Ranking Index*.

## Results and discussion

The following subsections discuss the effectiveness of the model as a tool to predict and explain variations in a bank’s *Risk Indicator*.

### Model performance as a predictive tool

**Fig. 4:**
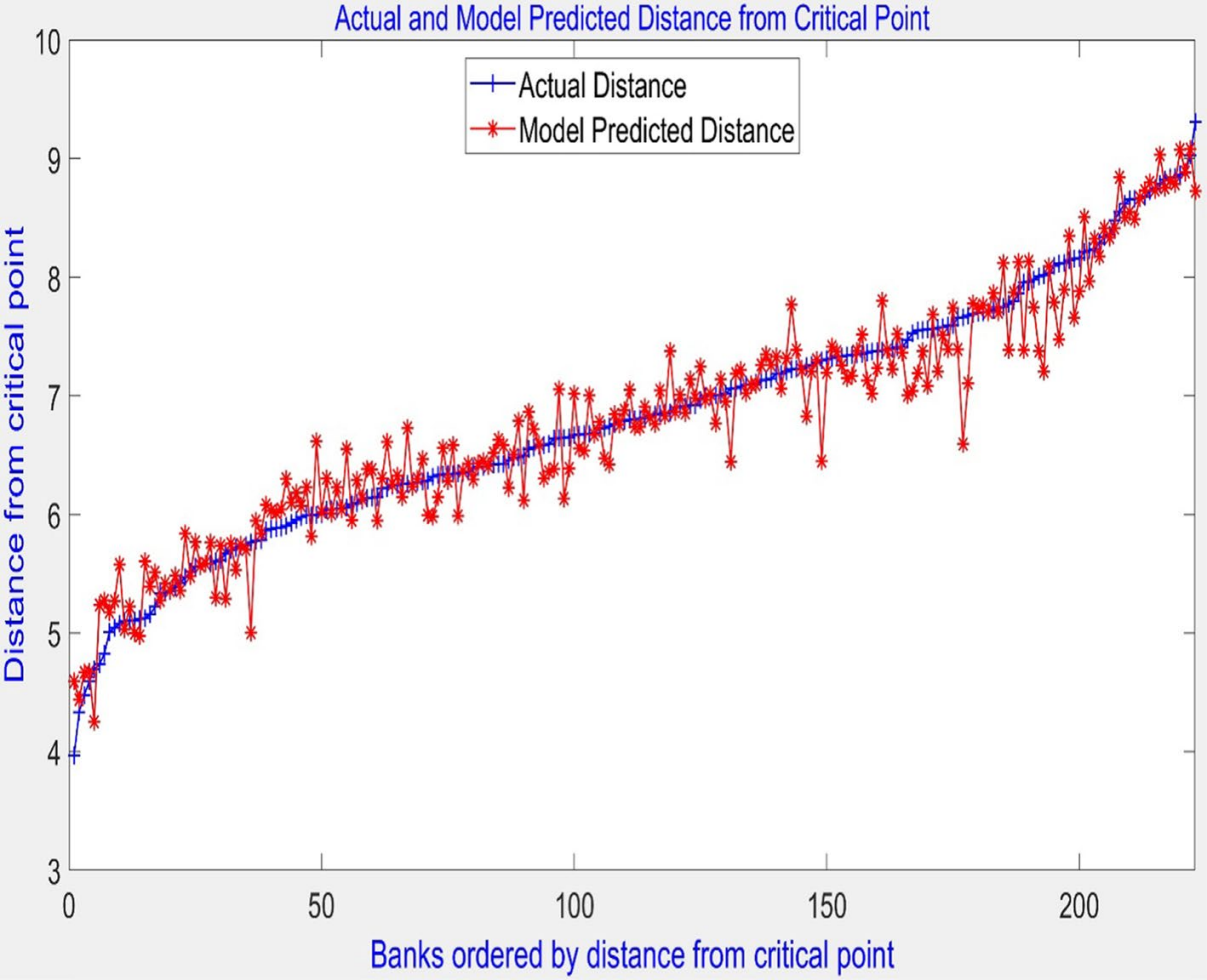
Actual and predicted risk ranking indicator (RRI)

**Fig. 5:**
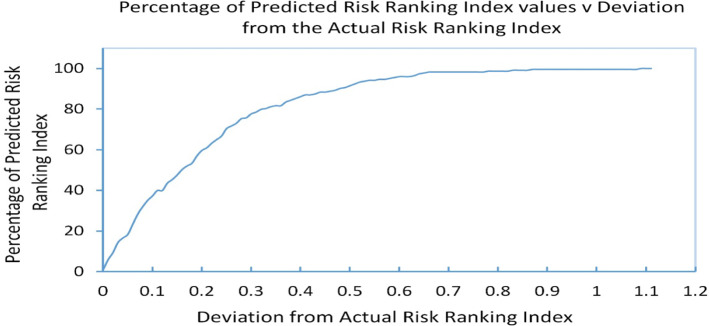
Percentage of predictions scores as a function of the deviation from the actual risk ranking index value

The model was trained using k-fold cross-validation with k = 10 to reduce overfitting. The root means square error (RMSE) of the model’s predictions obtained was 0.27. Figure 4 shows the actual RRI and model-predicted RRI. The percentage of predicted values that are within one RMSE of the true respective RRI value is 78% and 94% within two RMSEs. Figure 5 depicts the percentage of the RRI predicted scores as a function of the deviation from the actual RRI. We can see that all predicted RRIs fall within 0.75 of their respective true value, indicating that the model has implicitly captured the functional relationship between the financial ratios and the RRI. In the next step, the model is used to conduct sensitivity analysis to rank the individual risk ratios in terms of their importance in explaining variations in a bank’s risk position.

### Model explanatory performance

Sensitivity analysis was conducted to determine the causal importance of each input variable (financial ratio). Many methods have been proposed for neural network‐based sensitivity analysis (Cao et al. 2016). The partial derivative algorithm (Dimopoulos et al. 1995) and the input perturbation algorithm (Zeng and Yeung 2003) have been shown to have superior performance compared to other techniques (Gedeon. 1997; Wang et al. 2000). However, two major weaknesses can be found in the partial derivatives method. First, it cannot implement neural networks with non-differentiable activation functions and second, it is inadequate for calculating the magnitude effect of the input variable in output sensitivity assessment (Cheng and Yeung 1999).

In this work, we have chosen the perturbation method for the reasons cited above. This method perturbs a given input variable by adding noise while keeping all other inputs unchanged. The change ratio of the output variable regarding the perturbation in the input variable is calculated. The process is repeated for a number of different noise levels. The input variable with the most significant change ratio is the one that has the strongest explanatory effect on the output of the system being analyzed (Lamy 1996). The crucial issues, however, are: (i) selecting a reasonable index for measuring the change in the output and (ii) the range of input perturbation levels. Bai et al. (2011) has investigated several approaches to neural network sensitivity and showed that the formula given by Reddy et al. (2006) described in Eq. (9) measures correctly both the direction and magnitude of the sensitivity of a neural network output with respect to a perturbation in a particular input variable value:

9 $${S}_{j}= \frac{\Delta o}{\Delta {u}_{j}} ,$$

where,

10 $$\Delta o=\sum_{i=1}^{N}\left({\widehat{y}}_{i}-{y}_{i}\right),$$

11 $$\Delta u=\sum_{i=1}^{N}\left({\widehat{u}}_{i}-{u}_{i}\right)$$.

*S*_*j*_ is a sensitivity index of the output with respect to input *j,*

*N* is the number of input training vectors, $\widehat{y}, and y$ measures the network output with and without perturbation using the training data, and ${\widehat{u}}_{i}, and {u}_{i}$ are input variable *i* with and without noise, respectively.

To obtain an objective assessment of the sensitivity to perturbations in the input variables, the optimum range of input perturbation ratio should be determined (Bai et al. 2011). If the perturbation is overlarge, the sensitivity spectra may appear clipped. Generally, the farther a perturbation moves from the base case value, the less reliable the results become. However, if the perturbation is undersized, the sensitivity spectra may have no noise and sometimes no signal (Bai et al. 2011). Their study has found that a reasonable range of the input perturbation ratio is [− 20%, 20%] as there is no significant difference in the sensitivity of measurement within this range.

### Discussion of the findings

**Fig. 6:**
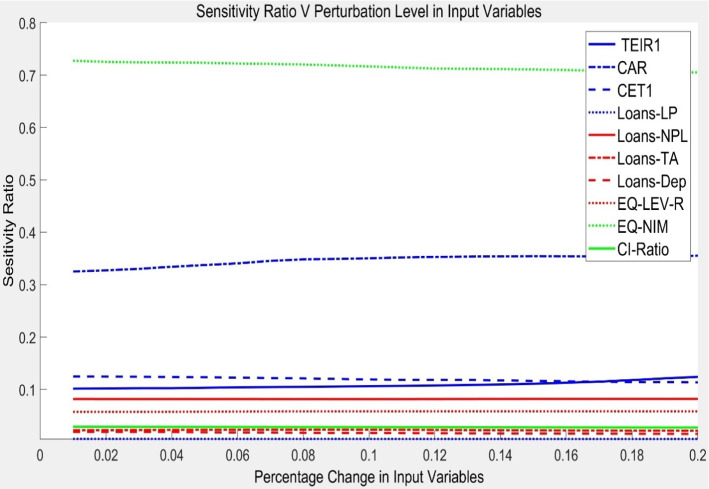
Sensitivity values of the financial ratios

After the Neuro-Fuzzy Model training had been completed, the sensitivity spectra values at increasing levels of input perturbation levels ranging from 0 to 20 % were calculated in steps of 0.01 according to formula (9) using training data. Table 3 and Fig. 6 show the sensitivity index of the model’s output for each input variable; these sensitivity index measurements indicate that by far Net Interest Margin Ratio (NIMR) is the most significant factor in explaining variations in bank risk position. NIM is an indicator of earnings’ quality and computed as the difference between the money that a bank is earning in interest on loans and the amount it is paying in interest on deposits. The justification for this high sensitivity is derived from variations in this ratio among the different banks in the different countries. NIMR is affected by factors of supply and demand for loans and the banking regulations that can increase or decrease the demand for deposit accounts and the demand for loans. This variable captures other sources of risk as it is an indicator of bank’s profitability and growth as well. This finding is consistent with previous studies such as; Saksonova, (2014) who finds that net interest margin is the most appropriate criterion for evaluating the effectiveness and stability of banks’ operations. Observe a negative relationship between NIM and the yield curve slope. Therefore, top ranking of earning quality risk is logical because it is very sensitive to the changes in loans and deposits.

- The second sensitivity index is the Capital Adequacy Ratio (CAR). It is noticed that the GCC banks are adequately capitalized and they meet the international criteria and many of the banks score higher than the target of all capital adequacy ratios. As evidenced by the model the CAR (0.3506) is more sensitive than the other two capital (CET1, 0.1189 and TEIR1, 0.1062). This result is consistent with Basel and Oudat (2020) who found a positive significant relationship between capital adequacy and banks’ performance in Bahrain. This finding is consistent with Basel and Oudat (2020), in which the capital risk is the most significant type of risk. The importance of capital adequacy risk is supported by Yang et al. (2020) who stated that banks with adequate capital are able to mitigate capital risk. The ranking of the CAR as a second important variable by this index is supported by its impact on different risk variables and other performance indicators. This was proved by El Ansari et al. (2019) when their study showed a positive association between CAR level and many of the performance indicators.
- Non-Performing Loans Ratio (NPLoans) is one of the measures of credit risk. The model shows that NPLoans with a sensitivity score of 0.0814 is somehow sensitive to the banking risk. Rajab and Sharma (2018) found the non-performing loan ratio to be one of the influential types of risk.
- The liquidity risk is measured by two ratios that consider the relationship of the banking loans to asset (LTAR 0.023) and to deposits (DepLoans, 0.0166). Both variables of liquidity score very low impact on the overall banking risk. This result is supported by Karamoy et al. (2020), who concluded that there is no significant impact of liquidity risk on banking performance. The GCC banks have low liquidity risks as they can be classified as conservative banks. This supports the findings of this model as it ranks loan to assets and loans to deposits at the end just before provisional loans ratio and explains their less sensitivity to the risk index.
  - The Provisional Loans Ratio (PLoans) appears to have no role to play as an explanatory variable of changes in a bank’s risk position. The loan loss provision is an allowance for uncollectible loans. Generally, the GCC banks haven’t much uncollectible loans. Therefore, this ratio is expected to have no real impact on banking risk index. It is important to note that these results are pertinent to the banks composing the dataset; however, the model can be used for any group of banks and financial ratios to analyze the relative strength of their financial position. Table 2 shows the relative ranking of the ten risk ratios based on their sensitivity index for an increase of 10% in the ten ratios.
  - Equity Leverage Ratio (ELEVR): Majority of the GCC banks are in a good capital position to meet the enhanced capital requirements of Basel III as most of them have a large portion of capital structure financed by equity. Therefore, the results of the model seem to be realistic as the ratio is not highly sensitive to risk and ranked in the sixth place with 0.0581.
  - The Cost Income Ratio (CIR): The model proves that the GCC banks are efficient in managing their cost in relation to the income they generate. A low sensitivity to risk of CIR (0.0278) is an indicator of low risk associated with this variable of operational risk group. The model results indicate that the performance of the banking sector from both operating expense reduction and revenue generation lead to a lower risk on the operational side. This ratio is classified as a lower weight ratio suggest that banks are well-capitalized and well-regulated with little variations among banks. BIS (2013) stated that 15% of banks funding are short-term in nature, although their cost-to-income are among the lowest ratios.
  - Both credit risk ratios are indexed at the end because 45% of GCC banks are Islamic banks. The existence of Islamic banks mitigates to a large extent the variables of credit risks. This ranking is consistent with Srairi (2013) who finds that Islamic banks display a lower exposure to credit risk as compared to the conventional ones.

**Table 3:** Financial ratios sensitivity index in influencing banks risk position

Financial ratio	Sensitivity index at 10% perturbation
Net Interest Margin Ratio (NIMR)	0.7168
Capital Adequacy Ratio (CAR)	0.3506
Common Equity Tier 1 Ratio (CET1)	0.1189
Tier 1 Equity Ratio (TEIR1)	0.1062
Non- Performing Loans Ratio (NPLoans)	0.0814
Equity Leverage Ratio (ELEVR)	0.0581
Cost Income Ratio (CIR)	0.0278
Loans Total assets Ratio (LTAR)	0.0230
Loan Deposit Ratio (DepLoans)	0.0166
Provisional Loans Ratio (PLoans)	0.0054

## Conclusions and recommendations

Although several studies have examined risk in emerging and advanced economies, studies on GCC banks are still limited. Over and above the study of all risk variables remains an unaddressed area of research, specifically in this part of the world. This paper has developed a banking risk index that incorporates the five main categories of banking risk with ten sub-variables measuring the risk components of each risk category. The study utilized data extracted from almost all the GCC banks (45 banks) over a 5-year period to develop the proposed risk index based on the Mahalanobis distance. ANFIS methodology based on a Segeno FIS model was used to build a prediction model to capture the relationship between the RRI and the various risk variables used. Using the ANFIS model, sensitivity analysis was conducted to determine the causal importance of each risk input variable. The main findings start with the development of a risk ranking index that measures the distance between the bank position, represented in terms of the 10 risk measures, from the critical values of these financial ratios stipulated by financial authorities and norms. Our findings indicate that a bank is in a sound financial position at the 99% and 90% confidence level if the value of the RRI is greater than 4.89 and 4.0, respectively. Sensitivity analysis of the functional model shows that by far Net Interest Margin Ratio (NIMR) is the most significant factor in explaining variations in bank risk position, followed by Credit Adequacy Ratio (CAR); while the Provisional Loans Ratio variable appears to have no role to play as an explanatory variable of changes in a bank’s risk position.

As a recommendation, the study shows that there are opportunities for future research to study the relationship between the different kinds of risks and banking performance. Further studies may be needed to segregate the risk of Islamic banks from conventional ones and develop separate indexes. The same study can be replicated at the country level to investigate the impact of certain local regulations on banking risk.

## Data Availability

The data that support the findings of this study are available from the corresponding author upon any request.
